# Addicted to love? Validity evidence for the Love Addiction Inventory — Brazilian version

**DOI:** 10.1186/s41155-025-00345-2

**Published:** 2025-04-30

**Authors:** Daniela Zibenberg, Jean Carlos Natividade

**Affiliations:** 1 Department of Psychology, Pontifícia Universidade Católica do Rio de Janeiro – PUC-Rio, Rio de Janeiro, Brazil; 2 Postgraduate Program in Psychology, Pontifícia Universidade Católica de Campinas – PUC-Campinas, Campinas, Brazil

**Keywords:** Scales, Dependency, Interpersonal relations, Obsessive behavior, Love addiction

## Abstract

**Background:**

The Love Addiction Inventory (LAI) is an instrument developed to assess the six dimensions of addiction to a romantic partner: salience, tolerance, mood modification, relapse, withdrawal, and conflict.

**Objective:**

The present study sought to adapt and provide validity evidence for the Brazilian version of the LAI.

**Method:**

An online questionnaire was completed by 1310 Brazilian adults who were in romantic relationships.

**Results:**

The data shows adequate reliability indices, validity evidence regarding the instrument’s internal structure, and validity based on the relationships among the variables. Notably, positive correlations between the LAI and Emotional Dependence Questionnaire and other addiction markers and negative correlations between the LAI and self-esteem were identified.

**Conclusion:**

These findings provide a helpful instrument for symptom tracking and investigating interventions for the treatment of love addiction in future research.

**Supplementary Information:**

The online version contains supplementary material available at 10.1186/s41155-025-00345-2.

## Introduction

The American Psychological Association defines addiction as a “… state of psychological and/or physical dependence on the use of drugs or other substances, such as alcohol, or on activities or behaviors. The term (…) can be applied to non-substance-related behavioral addictions, such as sex, exercise, and gambling” (APA, 2023). Although gambling disorder has been officially recognized based on evidence of the brain’s reward system activation and behavioral symptoms similar to those of other substance-related addictions (American Psychiatric Association, 2023), various behavioral addictions, including addiction to smartphones (Castellon et al., 2022), shopping (Lima et al., 2022), physical exercise (Restrepo et al., 2021), pornography (Taylor, 2020), and others, have been studied but are not yet considered official diagnosis in manuals like the *Diagnostic and Statistical Manual of Mental Disorders, Fifth Edition*, Text Revision (DSM- 5-TR; APA, 2023), or the International Classification of Diseases, 11 th revision (ICD- 11; World Health Organization, 2019) due to the lack of scientific evidence.

Daily vaping users reported higher levels of craving, valence, and sustained motivated attention for their beloveds in comparison to vaping, which suggests that love can be more addictive than an addictive substance, namely, vaping nicotine (Onyegbula et al., 2023). Love addiction (LA) is characterized as an independent mental disorder that may require psychotherapy or pharmacological treatment (Sanches & John, 2019). If unaddressed, it can lead to harmful and interpersonal violent behavior (Bradbury et al., 2024). Thus, it can negatively impact both the individual and those around them. Consequently, this study aimed to adapt and obtain validity evidence for a Brazilian version of an instrument measuring LA, the Love Addiction Inventory (LAI; Costa et al., 2021).

### Love addiction and behavioral addictions

LA is characterized by a pattern of behaviors directed toward a romantic partner that inevitably leads to negative consequences (Sussman, 2010). It is recognized as a behavioral addiction (e.g., Briggie & Briggie, 2015; Griffiths, 2019; Redcay & Simonetti, 2018; Sanches & John, 2019). Additionally, LA has been observed and studied in various cultures, such as Spain, the USA, Italy, and Brazil (e.g., Camarillo et al., 2020; Fisher, 2014; Orsolini et al., 2022; Sophia, 2014).

Some scholars are conducting studies to provide evidence that LA shares symptoms similar to other addictions and can be recognized as a non-substance addiction in psychiatric literature (e.g., Dineen & Dinc, 2024). There is substantial evidence that supports the idea of love as an addiction (Earp et al., 2017). However, LA remains a controversial concept, and researchers disagree on whether it should be defined narrowly or broadly. A meta-analysis revealed that the overall prevalence of behavioral addictions including internet, smartphone, gaming, social media, food, sex, exercise, gambling, and shopping addictions is 11.1% (Alimoradi et al., 2022). While some authors suggest that LA may affect nearly everyone in the world (e.g., Fisher et al., 2016), others estimate the LA prevalence around 3% (Sussman et al., 2011).

The narrow view posits that LA is identified by an individual’s abnormal behaviors in their pursuit of love, which disrupts daily functioning, hinders the development of healthy relationships, and results in clear negative consequences for themselves or others (Earp et al., 2017). From this perspective, the feelings, behaviors, and outcomes associated with love would either be minimal, manageable, or absent in individuals who do not experience LA.

The broader perspective suggests that loving someone can be akin to being addicted to them (Earp et al., 2017). In this context, romantic love shares several traits with addiction, including salience, craving, and neurobiological similarities (Bode & Kushnick, 2021). Individuals in the early stages of romantic love may exhibit signs similar to addiction, such as tolerance, withdrawal, and relapse (Fisher et al., 2016). Consequently, the authors propose that romantic love functions as a natural form of addiction that facilitates pair bonding and reproduction. While this love can have positive effects when it is reciprocated, non-toxic, and appropriate, it can be negative when it is rejected, toxic, or inappropriate. Hence, there is some criticism regarding the construct of LA, which is commonly addressed as a natural behavior similar to falling in love.

However, falling in love seems related to, yet distinct from LA, limerence, or infatuation (Lopez-Cantero, 2022). For example, cognitive task performance tends to decrease when individuals think about their beloved in the context of romantic love (Langeslag & Philippi, 2024). Still, this experience is typically described as enjoyable rather than intrusive or obsessive. This perspective differs from the broader viewpoint and contrasts with the definition of addiction, which involves the removal of something positive from life instead of adding something positive to it (Griffiths, 2005).

Similarly, a meta-analysis has shown that romantic love and addictive disorders differ in their neural representations (Yang et al., 2024). For example, the ventromedial prefrontal cortex shows greater activation during romantic love, whereas the posterior cingulate cortex is more active in addiction. This indicates that although both share a common neural foundation (e.g., the ventral anterior cingulate cortex), romantic love is associated with self-expansion and joyful growth, while addiction is associated with compulsive hedonism.

There are other critics of the concept of LA. For instance, viewing LA as a pathological condition can diminish love value and importance, reducing it to a set of chemical and neurobiological reactions (Özal et al., 2023). Furthermore, as with other behavioral addictions, there are concerns about the tendency to over-pathologize daily life activities (e.g., Billieux et al., 2015). This could lead to a standardization of love experiences. Undoubtedly, it is important to recognize that romantic relationships are not always a positive experience (Langeslag, 2024), and such experiences do not necessarily qualify as addiction or pathology. In this sense, it is noteworthy that treatment involves ethical considerations. It should only be pursued when there is suffering or dissatisfaction with the way one relates to others, along with the associated behaviors, thoughts, and emotions (Earp et al., 2017). Effective treatment should focus on promoting individual and relational well-being rather than imposing a specific way of relating to others.

Limerence is a construct similar to LA and is defined as obsessive and intrusive thoughts about a specific person (the limerence object), alongside a longing for romantic reciprocity (Tennov, 1998). The driving forces behind limerence are feelings of uncertainty and potential rejection. Once certainty is achieved, limerence decreases. Individuals who experience higher levels of loneliness or have unmet social needs and fantasized gratification may find it easier to develop limerence when they encounter someone who seems capable of fulfilling their desires and fantasies (Shaver & Hazan, 1985).

Individuals experiencing limerence report a need to ruminate and connect to the limerence object (Wyant, 2021). They often idealize the limerence object and exhibit behaviors such as constantly checking their social media and struggling to concentrate on tasks. They may experience mood swings based on interactions with that person, in which they feel relief when engaging in behaviors to increase contact and unpleasant emotional reactions when not in contact. Limerent individuals also spend a significant amount of time mind-wandering about their limerence object, and this mind-wandering is negatively associated with happiness and calmness and positively associated with sadness and anxiety (Evans, 2023).

Higher LA levels are positively associated with symptoms of anxiety and depression, cognitive deficits in memory and attention, and negatively associated with resilience (Giacobbe et al., 2024). Furthermore, individuals at risk for LA presented higher scores on emotion dysregulation and stress and inferior scores in self-concept clarity and caregiving behaviors (Cavalli et al., 2024). In addition, trait impulsivity (negative and positive urgency and sensation seeking), experiences of emotional and physical abuse during childhood (mediated by unbalanced family functioning), emotional and physical neglect, and vulnerable narcissism have been positively correlated with LA (Carone et al., 2024; Dineen & Dinc, 2024; Gori et al., 2024a, 2024b; Topino et al., 2024).

Furthermore, a variable often associated with LA is attachment. Several studies have found that LA is linked to insecure attachment (Cavalli et al., 2024; Rogier et al., 2024) and, more specifically, to fearful attachment (Topino et al., 2024). Additionally, LA is associated with the big five personality factors: agreeableness, consciousness, openness (negatively), and neuroticism (positively) (Gori et al., 2024a, 2024b). Furthermore, LA has been connected to problematic online dating app use and various addictions, including social media, sex, and pornography (Borello et al., 2023; Gori et al., 2024a, 2024b).

### Love addiction inventory

Griffith (2005) proposed a theoretical model that can be applied to miscellaneous behavioral addictions. This model comprises six dimensions: salience, tolerance, mood modification, relapse, withdrawal, and conflict. The Love Addiction Inventory (LAI; Costa et al., 2021) is an instrument based on Griffith’s model developed to assess addiction to love in the Italian population. The LAI consists of 24 items, with 4 items corresponding to each of the 6 factors that reflect the dimensions of this behavioral addiction model. In the context of LA, the salience dimension refers to a romantic partner’s central and primary importance in a person’s life, becoming the focus of their thoughts, behaviors, and feelings. Tolerance concerns the growing need to increase the time spent with or thinking about a romantic partner. Mood modification is defined as using the presence of a romantic partner or thoughts about them to cope with stress and reach emotional stability. Relapse refers to difficulty in stopping or reducing the time, attention, and thoughts dedicated to a romantic partner. Withdrawal corresponds to the signals and physical or psychological symptoms (e.g., irritability, anxiety, nausea, insomnia, tachycardia) experienced when an individual is emotionally or physically distant from their romantic partner. Finally, conflict relates to the influence of preoccupation with one’s romantic partner in daily activities such as those related to work, study, friendship, and leisure, resulting in a reduction or abandonment of interest and activities that were previously valued (Costa et al., 2021; Griffiths, 2005).

A total of 663 Italian adults participated in the original LAI elaboration study, 94.41% of whom were women (Costa et al., 2021). Although not all participants were currently in a romantic relationship, each had been in at least one relationship lasting at least 6 months. Data collection was conducted online. The data supported satisfactory psychometric evidence for the instrument, showing alpha coefficients of 0.94 for the total scale, 0.95 for the salience factor, 0.86 for the withdrawal factor, 0.89 for the tolerance factor, 0.91 for the mood modification factor, 0.77 for the relapse factor, and 0.85 for the conflict factor. These six factors explained 74% of the variance. Additionally, negative correlations with positive affect and positive correlations with negative affect were found. Clinical patients undergoing treatment for LA scored significantly higher in LAI scores in comparison to a control group (Borrello et al., 2022).

### Relationships between love addiction and emotional dependence and self-esteem

In a systematic review, Bution and Wechsler (2016) defined emotional dependence as the necessity for a romantic partner to achieve emotional stability. In the emotional dependence context, the romantic relationship is often the central part of the individual’s life. The Emotional Dependence Questionnaire (*Cuestionario de Dependência Emocional*; Hoyos & Arredondo, 2006) describes emotional dependence as the expectation that one’s romantic partner will fulfill a persistent pattern of unsatisfied affective needs (Castelló, 2005). Thus, emotional dependence encompasses separation anxiety and fear of losing one’s romantic partner, fear of loneliness, a constant need for affection, impulsivity and aggressiveness toward a potential end of the relationship, modifying plans to satisfy one’s romantic partner and spending more time with them, and seeking the attention and exclusivity of one’s romantic partner (Hoyos e Arredondo, 2006). Emotional dependence can also be viewed as a form of behavioral dependence (Camarillo et al., 2020; Olave et al., 2021). Hence, emotional dependence is a concept that closely resembles LA. By exploring the similarities between emotional dependence and LA, both of which are marked by addictive behaviors, it is possible to provide validity evidence for the Love Addiction Inventory (LAI). This examination can also assist in the selection of appropriate assessment tools and guide hypothesis development in future research, based on the instrument’s operational definition as well as its distinctions and overlaps.

Moreover, LA is related to self-esteem. Impairment in self-esteem may lead an individual to more strongly enrich the importance of a romantic relationship (salience) and engage more often in a problematic behavioral pattern. Conversely, low self-esteem may be a consequence of an addictive behavioral pattern. Accordingly, research indicates that self-esteem is negatively associated with LA (Feeney & Noller, 1990; Gori et al., 2023). Additionally, previous studies have shown a negative association between similar behavioral addiction in romantic relationships (i.e., pathological love, emotional dependence, and limerence) and self-esteem (e.g., Fônseca et al., 2020; Kim & Jeon, 2020; Neves & Hur, 2021). This correlational study not only provides validity evidence for the Brazilian version of the LAI but also suggests that understanding the negative relationship between LA and self-esteem can contribute to the development of future interventions aimed at modifying LA levels and provide evidence of the impact of LA on individuals’ self-esteem.

### Present study

Few instruments with psychometric robustness are available to assess LA, which creates even more barriers to its scientific study (Costa et al., 2021). The lack of a robust instrument to assess LA in Brazil also affects the ability to measure changes in LA levels after an intervention. Furthermore, scientific evidence regarding the treatment of LA is limited (Maglia et al., 2023; Reynaud et al., 2010; Sanches & John, 2019). Developing a LA instrument for Brazil can provide valuable information about LA in a Brazilian sample, which in turn can yield cross-cultural comparisons and contribute to gathering evidence to recognize LA as a non-substance-related addiction in diagnostic manuals. Additionally, it can increase the acknowledgment among professionals who treat individuals facing relational, affective, and love-related challenges and serve as a beneficial tool for future studies on these phenomena. In this way, the present study aimed to adapt the original version and obtain validity evidence for a Brazilian version of the LAI based on the characterization of its factor structure, reliability coefficients, and relationship with equivalent instruments and other variables.

Beyond the replication of the factorial structure of the original instrument, which consists of six factors, and in light of theoretical expectations and previous empirical findings (Camarillo et al., 2020; Feeney & Noller, 1990; Fônseca et al., 2020; Gori et al., 2023; Kim & Jeon, 2020; Neves & Hur, 2021; Olave et al., 2021), the following hypotheses were formulated:

- *H1: The scores from the six factors of the Brazilian version of the LAI will be positively correlated with the Emotional Dependence Questionnaire score.*
- *H2: Love addiction will be negatively correlated with self-esteem.*
- *H3: Love addiction, as assessed by the Brazilian version of the LAI, will be positively correlated with love addiction assessed by another measure, the Love Addiction Disorder Identification Test.*

## Methods

This study aimed to adapt the original version and obtain validity evidence for a Brazilian version of the LAI using a cross-sectional and correlational design.

### Participants

The sample in this study included 1.310 Brazilian adults with a mean age of 30.25 years (*SD* = 12.2). All participants were currently in a romantic relationship, with a mean length of 1811.2 days (4.96 years) (*DP* = 3131.6 days). A total of 18.3% of the participants were in nonmonogamous relationships, but participants were asked to consider only one partner when completing the questionnaire. Additionally, 29.5% of participants lived with their partner, and 11.8% had children together. More information on the sample can be seen in Table 1.

**Table 1:** Sample characteristics

Gender	Women	Men	Other/did not want to declare			
	84.3%	15.1%	0.60%			
Relationship type	Married	Common-law marriage	Engaged	Courtship	Going out	Other (e.g., friends with benefits)
	15.8%	7.6%	2.3%	44.4%	12.7%	17.2%
Sexual orientation	Heterosexual	Bisexual	Gay/lesbian	Other/did not want to declare		
	68.8%	24.3%	4.8%	2.2%		
Partner gender	Man	Woman	Other/did not want to declare			
	76.8%	22.7%	0.5%			
Race/ethnicity	White	Mixed race/brown	Black	Yellow	Indigenous	Did not want to declare
(*N* = 939)						
	68.1%	23.1%	6.2%	1.6%	0.2%	0.9%
Country region	South	Southeast	Midwest	Northeast	North	Currently living abroad
	10.1%	62.8%	5.3%	16.9%	3.2%	1.7%

### Instruments

The participants answered an online questionnaire composed of sociodemographic questions, such as questions about gender, age, relationship characteristics, and the scales described below.

#### Love Addiction Inventory — Brazilian version (LAI-BR)

This scale was adapted for Brazil in this study from the original version by Costa et al. (2021). It is a self-report scale composed of 24 items to assess the six factors of LA: salience, withdrawal, tolerance, mood modification, relapse, and conflict. Items are scored from 1 (never) to 5 (very frequently), based on how often respondents behave as described in each item. Item examples include “Feel anxious when you are not in the company of your partner” and “Stay with your partner to relieve stress.” The psychometric properties of this instrument are shown in the “Results” section.

#### Emotional dependence questionnaire (Fonsêca et al., 2020)

The Brazilian version of Hoyos e Arredondo’s (2006) scale comprises 23 items to assess the cognitive and psychological aspects of emotional dependence. Items are scored on a scale from 1 (completely false in me) to 6 (t perfectly describes me). An example item is “When my partner needs to go away for a few days, I feel anguish” and “I need to have one person with whom I will be more special to than others.” In the present study, the alpha coefficient for the instrument was 0.94.

#### Rosenberg self-esteem scale (Hutz & Zanon, 2011)

The Brazilian version of Rosenberg’s Self-Esteem Scale (1989) comprises 10 items used to assess self-esteem. Items are scored from 1 (totally disagree) to 4 (totally agree). Example items are “I feel I do not have much to be proud of” and “On the whole, I am satisfied with myself.”. In the present study, the alpha coefficient found was 0.91.

#### Love addiction disorder identification test (LADIT)

The LADIT, developed for this study, comprises 14 items specifically designed to assess love addiction disorder and negative relationship consequences. The items have three different response formats: three items are scored as “yes” or “no” (e.g., ‘Have you or someone else ever been injured as a result of your current relationship?’), four items are scored on a scale from 1 (“never”) to 11 (“every day of the week”) (e.g., ‘”How often have you failed to do what was normally expected of you because of your relationship?”), and seven items are scored from 1 (“strongly disagree”) to 7 (“strongly agree”) (e.g., “I fear that life without my partner would be boring, empty, and no fun”). The instrument’s items were inspired by the Brazilian versions of the Alcohol Use Disorder Identification Test (Babor et al., 2001; Santos et al., 2012), which assesses the frequency, adverse consequences of alcohol consumption, and dependency, as well as the Internet Addiction Test (Conti et al., 2012; Widyanto & McMurran, 2004), which assesses Internet addiction. Higher scores on the LADIT indicate greater levels of love addiction disorder and negative relationship consequences. The scale demonstrated a one-factor structure, a Comparative Fit Index (CFI) = 0.96, Tucker–Lewis index (TLI) = 0.95, root-mean-square error of approximation (RMSEA) = 0.059, weighted least square mean and variance adjusted (WLSMV) estimator, and an alpha coefficient of 0.83.

### Procedures

#### Translation

Inspired by the instrument adaptation guidelines of Beaton et al. (2000) and the cross-cultural adaptation considerations of Borsa et al. (2012), we followed a systematic process to ensure the accuracy and cultural relevance of the translated version of the LAI. Initially, three researchers with expertise in English independently translated the original scale, which was available in English and Italian, into Portuguese. A fourth researcher then carefully reviewed and synthesized these three translations into a single preliminary Portuguese version. Next, a back-translation process was conducted to assess the equivalence of this initial version to the original instrument. Two independent translators, fluent in the target languages, translated the Portuguese version back into English and Italian. These back-translated versions were subsequently compared with the original English and Italian versions of the scale. This comparison allowed two researchers to identify discrepancies and make minor adjustments to the wording of the Portuguese items, thereby improving their semantic and conceptual alignment with the original content. Following these refinements, the beta version of the Portuguese scale was tested in a pilot study involving 20 undergraduate and graduate psychology students. Participants were asked not only to respond to the items but also to provide feedback on any difficulties they encountered in understanding the wording or intent of the questions. Based on their responses and suggestions, final adjustments were made to ensure clarity and cultural appropriateness, leading to the final Brazilian version of the scale.

#### Ethical and data collection

All instruments and sociodemographic questions were presented in an online questionnaire. Participants were invited to complete the questionnaire through social media platforms (e.g., WhatsApp, Instagram, TikTok). The first page of the questionnaire included the free and informed consent term, along with information about the estimated time for completion, the study’s objectives, and assurances of voluntary and anonymous participation following the ethics committee guidelines. All participants provided informed consent before participating in the research. The Ethics in Research Committee Universidade Estácio de Sá approved this study under protocol number 5.903.034 (CAAE 66673522.2.0000.5284).

#### Data analysis

Primarily, the data were cleaned by including only the participants who answered the control items correctly. Control items were included in the questionnaire to guarantee that only data from participants who were aware of their answers were selected. Later, a correlation analysis between all the LAI-BR items was conducted. Skewness and kurtosis for each LAI item were calculated, and values ranged between − 0.155 and 0.970 for skewness and between − 1.42 and 0.136 for kurtosis. In sequence, confirmatory factor analyses were conducted to analyze the scale’s factor structure. The fit of the three models was obtained using a robust estimator suitable for ordinal data, the WLSMV estimator. We tested a six-factor model like the original scale version, a one-factor model, and a six-factor model with a second-ordered factor explaining them. These models were chosen based on the original scale from Costa et al. (2021), who also tested a six-factor and a second-order factor version for the original LAI and a one-factor version for the LAI short version. Cronbach’s alpha and McDonald’s omega coefficients were calculated for the adapted measure, and Cronbach’s alpha coefficients were calculated for the other measures used in this research. Alpha values above 0.70 were considered adequate (Nunnally & Bernstein, 1994). Subsequently, Pearson correlation analyses of the LAI-BR and the emotional dependence, self-esteem, and love addiction disorder measures, and the variables of relationship length, age, and meeting frequency were conducted. Additionally, ANOVA and Student’s *t* analyses were conducted to test the mean differences in the relationship status and habitation in the LAI-BR scores. The analyses were performed with the lavaan package (Rosseel, 2012), version 0.6.16, in R software, version 4.3.2 (R Core Team, 2021), and Jamovi software, version 2.3.26 (The Jamovi Project, 2022).

## Results

**Fig. 1:**
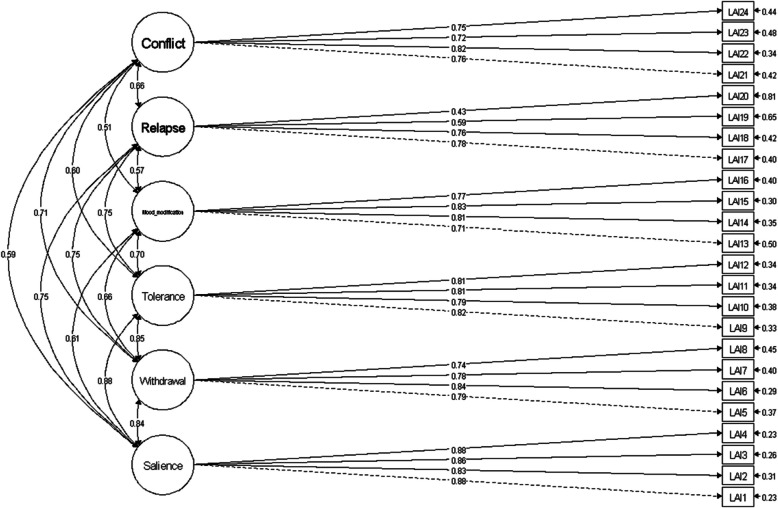
The six-factor model for the Love Addiction Inventory — Brazil. Note: *N* = 1306

Initially, evidence of validity based on the scale’s internal structure was sought. Confirmatory factor analyses were conducted for this purpose, testing the fit of three models using the WLSMV estimator: (1) a single-factor model, where a single factor explained all 24 items; (2) a second-order model, in which one second-order factor explained 6 first-order factors, each accounting for their respective items, similar to the original scale by Costa et al. (2021); and (3) a six-correlated-factors model, where 6 factors explained their respective items, also based on the original scale by Costa et al. (2021). Table 2 shows the results from the confirmatory factor analysis. The six-factor structure (Fig. 1), as described in the study by Costa et al. (2021), achieved a slightly better fit than the second-order factor structure, although both exhibited satisfactory fit indices. While the unifactorial model was the least suitable among the three models, it still demonstrated an adequate fit. Despite the six-factor and second-order factor structures having satisfactory fit indices, with the former slightly outperforming the latter, the six-factor structure aligned with the original instrument version. Consequently, per Costa et al. (2021), a composite total score for the LAI-BR was computed in addition to the scores for each factor.

**Table 2:** Adjustment indices for the three tested models

	One factor	One second-order factor and six factors	Six factors
*χ*^2^	3521.1	933.4	789.9
*df*	252	246	237
*p*	<.001	<.001	<.001
*χ*^2^/*df*	13.9	3.79	3.33
CFI	0.976	0.995	0.997
TLI	0.970	0.995	0.996
RMSEA	0.073	0.033	0.027
90% *CI* RMSEA	0.070–0.075	0.030–0.035	0.025–0.029

Weighted least-square mean and variance-adjusted (WLSMV) estimator; *CI* confidence interval

*N* = 1306

The LAI-BR factors exhibited correlations ranging from 0.51 to 0.88. The loading factors for the items ranged from 0.43 to 0.88. The LAI-BR items showed intercorrelations that ranged from 0.20 to 0.77. To estimate the reliability of the LAI-BR, alpha and omega coefficients were calculated: *α* = 0.92 and *Ω* = 0.92 for the salience factor, *α* = 0.87 and *Ω* = 0.87 for the withdrawal factor, *α* = 0.88 and *Ω* = 0.88 for the tolerance factor, *α* = 0.86 and *Ω* = 0.86 for the mood modification factor, *α* = 0.72 and *Ω* = 0.74 for the relapse factor, and *α* = 0.85 and *Ω* = 0.85 for the conflict factor.

In pursuit of validity evidence regarding relationships with other variables, correlation analyses were conducted between the factors of the LAI-BR and the factors of the measures of emotional dependence, self-esteem, and love addiction disorder and negative relationship consequences, as well as variables including meeting frequency, age, and relationship length. The results are shown in Table 3. All six factors of the LAI-BR and the composite total LAI score were positively correlated with measures of emotional dependence and love addiction disorder and negatively correlated with the measure of self-esteem. Furthermore, the LAI-BR factors and the total composite score were negatively correlated with the meeting frequency, age, and relationship length, except for the correlation between the mood modification factor and meeting frequency.

**Table 3:** Correlations between the Love Addiction Inventory — Brazilian version and the measures of emotional dependence, self-esteem, love addiction disorder, and demographic variables

	1	2	3	4	5	6	7	8	9	10	11	12
1. LAI — salience	—											
2. LAI — withdrawal	0.75**	—										
3. LAI — tolerance	0.80**	0.74**	—									
4. LAI — Mood Mod	0.54**	0.56**	0.60**	—								
5. LAI — relapse	0.62**	0.61**	0.61**	0.46**	—							
6. LAI — conflict	0.52**	0.61**	0.51**	0.43**	0.54**	—						
7. LAI	0.87**	0.87**	0.88**	0.74**	0.78**	0.73**	—					
8. Emo Dep, *N* = 1012	0.64**	0.74**	0.64**	0.55**	0.56**	0.63**	0.77**	—				
9. Self-esteem, *N* = 639	− 0.25**	− 0.34**	− 0.31**	− 0.35**	− 0.21**	− 0.31**	− 0.36**	− 0.42**	—			
10. LADIT, *N* = 1021	0.57**	0.69**	0.56**	0.47**	0.53**	0.65**	0.71**	0.74**	− 0.44**	—		
11. Meeting freq	− 0.21**	− 0.19**	− 0.29**	−.00	− 0.14**	−.06*	− 0.20**	− 0.15**	0.13**	− 0.15**	—	
12. Age	−.09*	−.06*	− 0.16**	− 0.29**	− 0.13**	−.08*	− 0.17**	− 0.14**	0.22**	− 0.10*	0.13**	—
13. Length	− 0.19**	− 0.15**	− 0.24**	− 0.23**	− 0.22**	− 0.10**	− 0.23**	− 0.20**	0.21**	− 0.21**	0.31**	0.64**

Note: *LAI* Love Addiction Inventory — Brazilian version, *Mood Mod* mood modification, *Emo Dep* emotional dependence, *LADIT* Love Addiction Disorder Identification Test, *Meeting Freq* meeting frequency,l Lengthrelationship length. When not mentioned, *N* = 1296

^**^p <.001

^*^p <.05

Furthermore, invariance across genders was established. In the configural invariance model, the CFI was 0.97, the TLI was 0.97, and the RMSEA was 0.041. For the scalar invariance model, a change in the CFI (ΔCFI) of − 0.002 was observed. For the metric invariance model, the ΔCFI was 0.000, and for the strict invariance model, the ΔCFI was also 0.000. These findings suggest that the differences in the LAI-BR responses were attributable to a latent trait rather than to issues with the instrument or its interpretation.

Additionally, mean differences in the LAI-BR were tested between relationship type and habitation (whether partners lived together or in separate houses). The results of the Student’s *t*-test and ANOVA are shown in Table 4.

**Table 4:** Mean differences between relationship type and habitation for theLove Addiction Inventory — Brazilian version

		Salience	Withdrawal	Tolerance	Mood Mod	Relapse	Conflict	LAI
	*N*	*M (SD)*	*M (SD)*	*M (SD)*	*M (SD)*	*M (SD)*	*M (SD)*	*M (SD)*
Married	305	2.46 (1.02)^ab^	2.12 (0.85)^ab^	2.53 (0.98)^ab^	2.20 (0.97)^ab^	2.10 (0.82)^ab^	2.29 (0.89)^ab^	2.28 (0.73)^ab^
Engaged	30	2.26 (1.12)^ab^	2.23 (0.95)^a^	2.78 (1.05)^a^	2.62 (0.93)	2.40 (1.07)^a^	2.05 (0.87)^a^	2.39 (0.81)^a^
Courtship	574	2.84 (1.09)^acd^	2.35 (1.00)^ad^	3.08 (1.03)^ad^	2.76 (1.02)^d^	2.62 (0.97)^ad^	2.50 (0.99)^d^	2.69 (0.82)^ad^
Going out	387	3.29 (1.15)^bcd^	2.83 (1.12)^bcd^	3.56 (1.04)^bcd^	2.68 (1.11)^d^	2.91 (1.05)^bcd^	2.56 (1.05)^d^	2.97 (0.89)^bcd^
Living together	385	2.48 (1.04)	2.17 (0.89)	2.56 (1.01)	2.37 (1.03)	2.23 (0.91)	2.29 (0.92)	2.35 (0.77)
Living apart	911	3.03 (1.13)	2.55 (1.08)	3.31 (1.05)	2.69 (1.06)	2.72 (1.01)	2.53 (1.01)	2.81 (0.85)

*Mood Mod*, mood modification. Tukey HSD post hoc test: ^a^significant difference from the going out group (*p* <.05). ^b^Significant differences from the courtship group. ^c^Significant differences from the engaged group. ^d^Significant differences from the married group

Concerning relationship type, all the ANOVA test results were significant: salience, *F*(3, 136.3) = 36.1, *p* < 0.001, *η*^2^ = 0.08; withdrawal, *F*(3, 137.2) = 30.6, *p* < 0.001, *η*^2^ = 0.07; tolerance, *F*(3, 1292) = 59.8, *p* < 0.001, *η*^2^ = 0.12; mood modification, *F*(3, 137.8) = 22.3, *p* < 0.001, *η*^2^ = 0.04; relapse, *F*(3, 135.7) = 45.6, *p* < 0.001, *η*^2^ = 0.08; conflict, *F*(3, 138.1) = 7.31, *p* < 0.001, *η*^2^ = 0.01; and the composite score, *F*(3, 136.8) = 44.0, *p* < 0.001, *η*^2^ = 0.09. For the salience and conflict factors, the going-out group scored higher, followed by the courtship, married, and engaged groups. For the withdrawal, tolerance, and relapse factors and the composite score, higher scores were found in the going out group, followed by the courtship, engaged, and married groups. For the mood modification factor, the courtship group had higher scores, followed by the going out, engaged, and married groups.

In addition, higher LA scores were found for partners living apart than for those living together for all factors: salience *t*(778.9) = − 8.41, *p* < 0.001, *d* = − 0.50; withdrawal *t*(864.8) = − 6.54, *p* < 0.001, *d* = − 0.38; tolerance *t*(748.8) = − 12.0, *p* < 0.001, *d* = − 0.73; mood modification *t*(738.5) = − 5.15, *p* < 0.001, *d* = − 0.31; relapse *t*(797.9) = − 8.61, *p* < 0.001, *d* = − 0.51; conflict *t*(793.9) = − 4.01, *p* < 0.001, *d* = − 0.24; and the composite score *t*(792.3) = − 9.33, *p* < 0.001, *d* = − 0.56.

## Discussion

The present study adapted the LAI and presented validity evidence of the LAI-BR. First, evidence regarding the instrument’s internal structure was obtained. A confirmatory factor analysis was conducted, and satisfactory indices were presented for the six-factor structure, similar to the original Italian instrument (Costa et al., 2021). Specifically, a CFI and TLI of 0.95 or higher and an RMSEA of 0.06 or below, as found for the LAI-BR, are considered good model-data fits (Hu & Bentler, 1999). Each factor retained all four expected items, along with the factor’s corresponding nomination: salience, withdrawal, tolerance, mood modification, relapses, and conflict. In addition, the reliability coefficients were found to be adequate. All the items were significantly intercorrelated, and the alpha and omega values ranged from 0.72 to 0.92, which are considered acceptable as long as they are 0.70 or above (Nunnally & Bernstein, 1994).

Evidence regarding relationships between equivalent measures and other variables is presented in sequence. All the LAI-BR factors were positively correlated with the emotional dependence and love addiction disorder measures, supporting hypotheses H1 and H3. The love addiction disorder identification test measure assesses love as an addiction, with alcohol and Internet items adapted to behaviors and feelings related to romantic relationships. Meanwhile, the emotional dependence measure assesses the reliance on a romantic partner to meet affective needs, considering aspects such as separation anxiety, fear of abandonment, and seeking a partner’s attention (Castelló, 2005; Fônseca et al., 2020; Hoyos & Arredondo, 2006). These positive and significant correlations indicate validity evidence based on the relation with other variables for the LAI-BR, demonstrating its relationship with equivalent measures and similar constructs.

LA was negatively correlated with self-esteem. This corroborates hypothesis H2, which predicted this relationship based on previous studies that indicated a negative relationship between LA and similar conditions, including emotional dependence and pathological love (different from erotomania, the study defined pathological love as an act of caring for and giving attention to one’s partner in greater quantity than one would like; for a precise definition, see Sophia, 2014) with self-esteem (Fônseca et al., 2020; Gori et al., 2023; Neves & Hur, 2021). According to these findings, low self-esteem may lead to addictive patterns in romantic relationships. Hence, future studies could test whether interventions aimed to boost self-esteem can reduce LA levels. This suggests that low self-esteem could be considered a risk factor for developing LA. Additionally, LA may also have negative consequences on individuals’ well-being, potentially harming their self-esteem.

The finding that LA is negatively associated with self-esteem and positively associated with emotional dependence suggests that higher levels of LA may lead to greater harm for individuals. Previous studies have already demonstrated a link between LA and impairment (e.g., Cavalli et al., 2024; Dineen & Dinc, 2024; Evans, 2023). This aligns with the narrow view, which claims that extreme behavior in the quest for love is associated with harm (Earp et al., 2017). Thus, LA represents a condition that can cause significant suffering and harm and may even be as addictive—if not more so—than some substance addictions (Onyegbula et al., 2023).

However, the experience of romantic love shares several characteristics with LA, and it is a misconception to assume that romantic love only has positive outcomes on people’s lives (Bode & Kushnick, 2021; Langeslag, 2024). For example, romantic love can provoke jealousy, grief, or stress. Thus, while these findings alone are inconclusive for firmly endorsing either the broad or narrow perspective, the evidence of harm aligns with Earp et al. (2017), who ethically propose addressing harm regardless of diagnostic conditions. This is particularly important because individuals who are simply “in love” and experiencing impairment because of it may not necessarily perceive these harms as problematic (Langeslag & Philippi, 2024).

Furthermore, the salience, withdrawal, tolerance, conflict, and relapse factors were negatively correlated with meeting frequency. This means that the lower the frequency of meetings with a partner, the higher the LA levels. A lower meeting frequency may create feelings of uncertainty in the relationship due to less investment or long distance, which, in turn, can contribute to increased LA. In addition, it is also possible that higher levels of LA lead to a decreased desire from the partner to meet, resulting in reduced meeting frequency and likely affecting the overall relationship quality.

All the LAI-BR factors were negatively correlated with age and relationship length. These findings corroborate the hypothesis that LA is associated with uncertainty, in which individuals in longer relationships or older individuals with more relationship experience may feel more confident in their partnerships and consequently present lower levels of LA. Thus, LA seems more prevalent in younger individuals and shorter relationships. Previous studies have shown a greater prevalence of romantic love characterized by obsession in short-term relationships (Maglia et al., 2023), as well as higher levels of pathological love among individuals with shorter relationships (Neves & Hur, 2021), and higher levels of pathological love and LA in younger individuals, in comparison to individuals in the condition of low LA or without pathological love (Giacobbe et al., 2024; Sophia, 2014).

Moreover, mean differences were calculated for relationship type and habitation. LA scores were higher for those living apart than for those living together. Previous studies also revealed a greater prevalence of pathological love in people living without their partners (Sophia, 2014). Furthermore, greater LA means were observed in the going out and courtship groups, whereas lower means were found for the engaged and married groups. These findings further support the idea that LA is associated with investment and uncertainty in relationships.

The married and engaged groups, as well as the living together group, had more committed relationships than the courtship, going out, and living apart groups. This greater level of commitment was associated with lower levels of LA. However, it is also possible that living with a partner reduces LA characteristics, such as a sense of urgency to meet, withdrawal symptoms, and the need to spend more time with one’s partner, because partners are likely meeting frequently and consistently. In this sense, engaged and married couples mostly live together. In addition, the mood modification factor was the only factor for which the courtship group scored higher than the going out group. This reflects the greater intimacy that partners share in courtship compared to going out. During courtship, an individual’s partner often serves as part of their support network and helps with coping, while in going-out partners are still trying to get to know each other. Although mood modification should ideally consider the use of the partner as a source of emotional regulation, similar to a drug that alleviates distress, it is possible that part of partner coping may be reflected in the responses to the items.

These findings, which show that uncertainty and investment are associated with LA, align with the limerence theory (see Tennov, 1998). According to this theory, experiences of rejection and uncertainty in a relationship intensify obsession and rumination, with the ultimate goal being reciprocity (Tennov, 1998). Individuals in more committed relationships, such as those engaged, married, living together, or meeting frequently, can receive more precise signals of reciprocity and experience greater certainty. Consequently, this clarity reduces the behaviors, thoughts, and emotions typically associated with LA.

LA is associated with harm and impairment, such as anxiety, depression, and stress (Cavalli et al., 2024), and can provoke interpersonal violence (Bradbury et al., 2024). The literature concerning this type of addiction is still scarce, and little is known about its prevention and treatment (Maglia et al., 2023; Reynaud et al., 2010; Sanches & John, 2019). Among the few instruments available to assess LA, the LAI is the only instrument based on a robust model of addiction that can apply to any behavioral addiction (Griffiths, 2005). In addition, the Brazilian version of the LAI facilitates cross-cultural comparisons, tracking LA symptoms, and use in future studies.

The present study has several limitations that could be addressed in future studies. First, the instrument’s temporal stability was not tested, and there is no consensus regarding the minimum period for which LA symptoms must be present to be considered harmful. Strategic foresight involves collecting longitudinal data and verifying temporal stability and symptom persistence. Some limitations of the original LAI study were maintained in the present study, such as the reliance on self-reported measures and a greater proportion of female participants. This gender imbalance requires caution when interpreting the results. In addition, men are typically expected to be self-sufficient and autonomous, which impedes greater intimacy, connection, or care (Elliott et al., 2022). Hence, men who agree to take a survey about LA may be more open to talking about feelings or admitting their lack of independence, potentially leading to bias in the data.

Furthermore, clinical patients were not included in this study, and because love addiction disorder is not an official diagnosis, it was not possible to ask the participants if a health professional had previously diagnosed them with this condition. Including clinical patients would enable the use of statistical analysis, such as ROC curves on dimensional data from the LAI-BR, to calculate the test’s sensitivity and specificity, thereby establishing a cutoff point for diagnosis (e.g., Hoo et al., 2017). In addition, the love addiction disorder identification test was utilized as an instrument for providing validity evidence based on the relationship with other variables. While we presented some preliminary validity evidence for this instrument, it has not been previously published or formally validated. Finally, future studies could explore the relationships between LA and other variables, such as violence and abuse in relationships, and variables that contribute to the development of healthy, gentle, and genuinely satisfying relationships.

### Implications

The findings from this study suggest that LA is positively associated with emotional dependence and negatively associated with self-esteem, meeting frequency, age, and relationship length in a Brazilian sample. These results build on existing evidence of LA linking to limerence, uncertainty, and investment in the romantic relationship. Additionally, the negative association with self-esteem should be viewed as evidence of individual suffering and harm, in line with the narrow perspective on LA (Earp et al., 2017).

It is important to formulate interventions that prevent negative outcomes associated with LA. For example, Chen and Hou (2025) have applied a gamification mechanism in relationship education to enhance the knowledge of adult students regarding LA-related issues, and Özal et al. (2023) have proposed implementing individual and collective preventive strategies to mitigate the consequences of relationship addiction. These strategies include fostering secure attachment and enhancing self-esteem, as well as promoting healthy relationships through mass media and school education. The authors also highlight the importance of developing validated measures with a strong theoretical basis for accurate diagnosis and treatment of relationship addiction. Thus, the present study provided a Brazilian version of the Love Addiction Inventory, a worldwide used instrument with a strong theoretical basis to assess LA.

This enables cross-cultural comparisons on LA, to have data on LA Brazilian prevalence, and to explore the relationships between LA and other variables, both those already tested in other cultures such as support (Borrello et al., 2022), and new variables that may be more relevant in the Brazilian context, such as gender roles and sociosexuality. Additionally, the availability of the LAI adapted for Brazil also facilitates the formulation and testing of treatment and prevention interventions for LA, aimed at implementing powerful and effective interventions to reduce the potential harms associated with LA.

## Conclusions

This study presented validity evidence and reliability indices of the LAI-BR. Validity was based on the instrument’s internal structure and relations with self-esteem, emotional dependence, and love addiction disorder measure. This study provided information regarding LA within a Brazilian sample, contributing to cross-cultural comparisons and establishing a robust instrument for assessing LA. The instrument can be used in future studies, as well as for symptom screening and tracking. The scientific investigation of love addiction is necessary to gather evidence and determine whether or not it should qualify as a diagnosis. Although further research is needed to confirm this, this study represents another step forward.

## Supplementary Information

Supplementary Material 1.

## Data Availability

The datasets used and/or analyzed during the current study are available from on reasonable request.

## Abbreviations

CFI: Comparative Fit Index
CI: Confidence interval
DSM- 5-TR: *Diagnostic and Statistical Manual of Mental Disorders, Fifth Edition*, Text Revision
ICD- 11: International Classification of Diseases, 11 th revision
LA: Love addiction
LADIT: *Love Addiction Disorder Identification Test*
LAI: Love Addiction Inventory
LAI-BR: Love Addiction Inventory — Brazilian version
RMSEA: Root-mean-square error of approximation
TLI: Tucker–Lewis index
WLSMV: Weighted least square mean and variance adjusted
