# Supplementary material for: Addicted to love? Validity evidence for the Love Addiction Inventory — Brazilian version
Source: Psicol Reflex Crit. 2025 Apr 30;38:13. doi: 10.1186/s41155-025-00345-2 (PMC12044147; doi:10.1186/s41155-025-00345-2)
Supplement: Supplementary file 1 — Supplementary Material 1. [file 41155_2025_345_MOESM1_ESM.docx]

**Love Addiction Inventory - Brazil (LAI-BR; Zibenberg & Natividade, 2025)**

Assinale o quão frequente você… *(Signalize how often do you…)*

|  | Nunca  *(Never)*  1 | Raramente  *(Rarely)*  2 | Às vezes  *(Sometimes)*  3 | Frequentemente  *(Often)*  4 | Muito frequentemente  *(Very often)*  5 |
| --- | --- | --- | --- | --- | --- |
| 1. Sente a necessidade urgente de encontrar com seu(sua) parceiro(a). *(Feel the urgent need to meet with your partner)* | 1 | 2 | 3 | 4 | 5 |
| 2. Tenta urgentemente ver seu(sua) parceiro(a). *(Try to urgently see your partner)* | 1 | 2 | 3 | 4 | 5 |
| 3. Sente a necessidade urgente de estar com seu(sua) parceiro(a). *(Feel the urgent need to be with your partner)* | 1 | 2 | 3 | 4 | 5 |
| 4. Sente o desejo urgente de querer estar na companhia de seu(sua) parceiro(a). *(Feel the urgency of wanting to be in the company of your partner)* | 1 | 2 | 3 | 4 | 5 |
| 5. Se sente agitado quando não está com seu(sua) parceiro(a). *(Feel agitated when you are not with your partner)* | 1 | 2 | 3 | 4 | 5 |
| 6. Se sente ansioso(a) quando não está na companhia de seu(sua) parceiro(a). (*Feel anxious when you are not in the company of your partner)* | 1 | 2 | 3 | 4 | 5 |
| 7. Se sente deprimido na ausência de seu(sua) parceiro(a). (*Feel depressed in your partner’s absence)* | 1 | 2 | 3 | 4 | 5 |
| 8. Se sente abandonado(a) quando não está com seu(sua) parceiro(a). *(Feel abandoned when you are not with your partner)* | 1 | 2 | 3 | 4 | 5 |
| 9. Sente a necessidade de passar cada vez mais tempo com seu(sua) parceiro(a) para sentir prazer. *(Feel the need to increase the amount of time you spend with your partner to experience pleasure)* | 1 | 2 | 3 | 4 | 5 |
| 10. Sente a necessidade de aumentar o número de encontros com seu(sua) parceiro(a) para se sentir feliz. *(Feel the need to increase the number of meetings with your partner to feel happy)* | 1 | 2 | 3 | 4 | 5 |
| 11. Sente a necessidade de aumentar o número de encontros com seu(sua) parceiro(a) para se sentir satisfeito(a). *(Feel the need to increase the number of meetings in the company of your partner to feel satisfied)* | 1 | 2 | 3 | 4 | 5 |
| 12. Sente a necessidade de passar mais tempo com seu(sua) parceiro(a) para se sentir relaxado(a). *(Feel the need to increase the time spent together with your partner to feel relaxed)* | 1 | 2 | 3 | 4 | 5 |
| 13. Fica com seu(sua) parceiro(a) para aliviar o estresse. *(Stay with your partner to relieve stress)* | 1 | 2 | 3 | 4 | 5 |
| 14. Passa tempo com seu(sua) parceiro(a) para esquecer do seu sofrimento. *(Spend time with your partner to forget about your suffering)* | 1 | 2 | 3 | 4 | 5 |
| 15. Passa tempo com seu(sua) parceiro(a) para evitar ficar de mau humor. *(Spend time with your partner to avoid being in a bad mood)* | 1 | 2 | 3 | 4 | 5 |
| 16. Passa tempo com seu(sua) parceiro(a) para aliviar seus sentimentos negativos. *(Spend time with your partner to reduce your negative feelings)* | 1 | 2 | 3 | 4 | 5 |
| 17. Não consegue passar menos tempo com seu(sua) parceiro(a). *(Fail to spend less time with your partner)* | 1 | 2 | 3 | 4 | 5 |
| 18. Não consegue diminuir a duração dos encontros com seu(sua) parceiro(a). (*Fail in reducing the duration of meetings with your partner)* | 1 | 2 | 3 | 4 | 5 |
| 19. Falha em evitar encontrar seu(sua) parceiro(a). *(Fail to avoid meeting with your partner)* | 1 | 2 | 3 | 4 | 5 |
| 20. Não consegue reduzir o tempo que passa com seu(sua) parceiro(a). *(Not reduce the time spent with your partner)* | 1 | 2 | 3 | 4 | 5 |
| 21. Abandona seus hobbies para estar com seu(sua) parceiro(a). *(Abandon your hobbies to be with your partner)* | 1 | 2 | 3 | 4 | 5 |
| 22. Abandona suas atividades sociais e recreativas para estar se relacionando com seu(sua) parceiro(a). *(Leave your recreational and social activities to be in a relationship with your partner)* | 1 | 2 | 3 | 4 | 5 |
| 23. Por vezes, deixa de lado compromissos familiares e sociais devido ao relacionamento com seu(sua) parceiro(a). *(Discard your family and social commitments due to the relationship with your partner)* | 1 | 2 | 3 | 4 | 5 |
| 24. Negligencia seu tempo de estudo ou trabalho para estar se relacionando com seu(sua) parceiro(a). *(Neglect time studying or working to be in the relationship with your partner)* | 1 | 2 | 3 | 4 | 5 |
